# Simultaneous upgrading of biomass-derived sugars to HMF/furfural via enzymatically isomerized ketose intermediates

**DOI:** 10.1186/s13068-019-1595-4

**Published:** 2019-10-26

**Authors:** Wei Wang, Ashutosh Mittal, Heidi Pilath, Xiaowen Chen, Melvin P. Tucker, David K. Johnson

**Affiliations:** 1 Biosciences Center, National Renewable Energy Laboratory, Golden, CO 80401 USA; 2 National Bioenergy Center, National Renewable Energy Laboratory, Golden, CO 80401 USA

**Keywords:** Glucose, Xylose, Enzymatic isomerization, Ketose, HMF, Furfural, Dehydration

## Abstract

**Background:**

Recently, exploring fermentative or chemical pathways that convert biomass-derived sugars to fuels/chemicals has attracted a lot of interest from many researchers. We are investigating a hydrocarbon pathway from mixed sugars via 5-hydroxymethyl furfural (HMF) and furfural intermediates. To achieve this goal, we must first convert glucose and xylose to HMF and furfural in favorable yields. Current processes to produce HMF/furfural generally involve the use of acid catalysts in biphasic systems or solvents such as ionic liquids. However, the yield from transforming glucose to HMF is lower than the yield of furfural from xylose.

**Results:**

In this study, we present an efficient chemical pathway simultaneously transforming glucose and xylose to HMF and furfural via ketose intermediates, i.e., fructose and xylulose, which were generated from glucose and xylose via enzymatic isomerization. In the enzymatic isomerization, by adding sodium borate to complex with the ketoses, xylose conversion reached equilibrium after 2 h with a conversion of 91% and glucose conversion reached 84% after 4 h. By enzymatically isomerizing the aldoses to ketoses, the following dehydration reactions to HMF and furfural could be performed at low process temperatures (i.e., 110–120 °C) minimizing the side reactions of the sugars and limiting the degradation of furfurals to humins and carboxylic acids. At 120 °C, pH 0.5, and 15 min reaction time, mixed ketose sugars were converted to HMF and furfural in yields of 77% and 96%, respectively (based on starting aldose concentrations).

**Conclusion:**

Taken together, our results demonstrate that this combined biological and chemical process could be an effective pathway to simultaneously convert biomass-derived glucose and xylose to HMF and furfural, for use as intermediates in the production of hydrocarbons.

## Background

Lignocellulosic biomass is considered to be the only sustainable resource with the potential to deliver renewable fuels and biobased chemicals [1, 2]. In the past decade, exploring chemical or fermentative pathways to convert biomass-derived sugars to fuels/chemicals has attracted a lot interest from researchers. It is well known that glucose and xylose are the two main sugars making up the carbohydrates in biomass feedstocks, therefore, our overall goal was to convert mixed pentose and hexose containing process streams into hydrocarbons for blending into jet and diesel fuels.

Current processes to produce HMF/furfural generally involves the use of acid catalysts in aqueous media or solvents such as ionic liquids and the chemical transformation has mostly utilized only one type of sugar, i.e., glucose or xylose, [3–7] rarely both sugars simultaneously. To maximize the utilization of sugar components in biomass feedstock, we must develop pathways for converting mixed pentoses and hexoses into hydrocarbons. However, it is well known from the literature that the conditions needed for converting glucose to HMF are much more severe than those required for converting xylose to furfural, such that if these conditions were applied to a mixture of pentose and hexose sugars it is likely that any furfural produced would be substantially degraded. Considering the large amount of glucose in biomass, an effective way to co-transform glucose/xylose to HMF/furfural with high yields is important. In this context, to overcome the obstacles for utilizing mixed sugars in a dehydration/condensation/hydrodeoxygenation pathway, we must convert the C6 sugars into HMF under much less severe conditions than have typically been used.

It is also well known that ketoses can be dehydrated under much less severe conditions than aldoses. Chemical transformation of ketose sugars, i.e., fructose and xylulose, has been reported as an efficient pathway to HMF/furfural [8–13]. In those reactions, ketose sugars were produced by the isomerization of aldose sugars, i.e., by converting glucose and xylose to fructose and xylulose, respectively, using enzymes or Lewis acids. Compared to chemical isomerization, enzymatic reaction is more specific and milder. It is well known that the biochemical conversion of glucose to fructose has been performed industrially for the production of high fructose corn syrup using immobilized enzymes such as Sweetzyme IT (Novozyme) or Gensweet IGI (Genencor) [14–16]. As the glucose isomerase is also functional on other aldose sugars, this enzyme can also be used for the isomerization of xylose to xylulose [17, 18]. Thus, simultaneous isomerization of two aldose sugars can be achieved using one enzyme. In this study, we used Sweetzyme, the commercially available enzyme produced from *Streptomyces* species to catalyze the isomerization reaction of d-glucose and d-xylose. In industry for production of high-fructose syrup, by using a process design of multiple reactors connected in series or parallel, the enzyme bed of Sweetzyme IT can have a lifetime of 1050 h [19]. The optimum pH for stable operation of the isomerase is pH 7.5 at 60 °C, which were the parameters used in this study. Here we report on the enzymatic isomerization of pure glucose and xylose, the enzymatic isomerization of the sugars in pretreated biomass hydrolysate slurry, and the subsequent dehydration of the ketose sugars to HMF and furfural.

## Materials and methods

### Materials

Chemicals in this study glucose (≥ 99.5%), xylose (≥ 99%), and sodium tetraborate were purchased from Sigma-Aldrich. Immobilized glucose isomerase Sweetzyme was also purchased from Sigma-Aldrich. Xylulose was purchased from Carbosynth Ltd. (Berkshire, UK). A process-relevant glucose and xylose rich sugar hydrolyzate was obtained from corn stover going through either deacetylation and mechanical refining (DMR) pretreatment [20] or dilute acid pretreatment [21] followed by enzymatic saccharification of the pretreated corn stover.

### Enzymatic isomerization of sugars

Sugars were prepared in Tris buffer (50 mM, pH 7.5). The reaction volume was 10 mL in 125 mL flasks. Borate was added to the sugar solution at a molar ratio of 2:1 (sugar:borate) and immobilized enzyme was added at a loading of 0.5 g/g sugar unless otherwise noted. The isomerization reaction was conducted for 6 h at 60 °C at a shaking speed of 130 rpm. Samples were taken at 0 h, 1 h, 2 h, 4 h and 6 h for sugar analysis via high-performance liquid chromatography (HPLC) on an acid column or with the Dionex system as described in “Analysis of sugars” section below. With DMR hydrolysate as substrate, original undiluted hydrolysate (~ 90 g/L glucose and 60 g/L xylose) was used with no buffering. Borate and enzyme were added at the same ratio as with pure sugars. The glucose conversion and fructose yields were calculated using Eqs. 1 and 2 below, where Glu_in_ and Glu_fin_ are the amount of glucose, Fru_in_ and Fru_fin_ are the amount of fructose, initially and after the isomerization reaction. The xylose conversion and xylulose yields were calculated using Eqs. 3 and 4 below, where Xyl_in_ and Xyl_fin_ are the amount of xylose, Xylu_in_ and Xylu_fin_ are the amount of xylulose, initially and after the isomerization reaction.

1 $${\text{Glucose}}\;{\text{conversion }}\left( \% \right) = \frac{{{\text{Glu}}_{\text{in}} - {\text{Glu}}_{\text{fin}} }}{{{\text{Glu}}_{\text{in}} }} \times 100$$

2 $${\text{Fructose}}\;{\text{yield }} \left( \% \right) = \frac{{{\text{Fru}}_{\text{fin}} - {\text{Fru}}_{\text{in}} }}{{{\text{Glu}}_{\text{in}} }} \times 100$$

3 $${\text{Xylose}}\;{\text{conversion }} \left( \% \right) = \frac{{{\text{Xyl}}_{\text{in}} - {\text{Xyl}}_{\text{fin}} }}{{{\text{Xyl}}_{\text{in}} }} \times 100$$

4 $${\text{Xylulose}}\;{\text{yield }} \left( \% \right) = \frac{{{\text{Xylu}}_{\text{fin}} - {\text{Xylu}}_{\text{in}} }}{{{\text{Xyl}}_{\text{in}} }} \times 100.$$

### Dehydration reaction experiments

Acid-catalyzed sugar dehydration experiments were performed in batch mode using a Discover S-Class microwave-heated reactor (CEM Explorer, Matthews, NC). Sugar dehydrations were conducted as described in previous work [7]. The reactions were run at a temperature of 120 °C and pH 0.5 using HCl as the catalyst. Dioxane was used in a volume ratio to aqueous of 2:1. All samples were run in triplicates at various reaction times within each series of experiments. The glucose conversion and HMF yields are calculated following Eqs. 5 and 6 below, where Glu and HMF are the amounts of glucose and HMF present in each phase (organic and aqueous) initially (in) and after the dehydration reaction (fin). The xylose conversion and furfural yields are calculated following Eqs. 7 and 8, where Xyl and Fur are the amounts of xylose and furfural present in each phase (organic and aqueous) initially (in) and after the dehydration reaction (fin).

5 $${\text{Glucose}}\;{\text{conversion }} \left( \% \right) = \frac{{{\text{Glu}}_{\text{in}} - {\text{Glu}}_{\text{fin}} }}{{{\text{Glu}}_{\text{in}} }} \times 100$$

6 $${\text{HMF}}\;{\text{yield }} \left( {\% {\text{mol}}/{\text{mol}}} \right) = \frac{{({\text{HMF}}_{{{\text{org}},{\text{fin}}}} + {\text{HMF}}_{{{\text{aq}},{\text{fin}} }} ) - ({\text{HMF}}_{{{\text{org}},{\text{in}}}} + {\text{HMF}}_{{{\text{aq}},{\text{in}}}} )}}{{{\text{Glu}}_{\text{in}} }} \times \frac{180}{126}$$

7 $${\text{Xylose}}\;{\text{conversion}} \left( \% \right) = \frac{{{\text{Xyl}}_{\text{in}} - {\text{Xyl}}_{\text{fin}} }}{{{\text{Xyl}}_{\text{in}} }} \times 100$$

8 $${\text{Furfural}}\;{\text{yield }} \left( {\% {\text{mol}}/{\text{mol}}} \right) = \frac{{({\text{Fur}}_{{{\text{org}},{\text{fin}}}} + {\text{Fur}}_{{{\text{aq}},{\text{fin}} }} ) - ({\text{Fur}}_{{{\text{org}},{\text{in}}}} + {\text{Fur}}_{{{\text{aq}},{\text{in}}}} )}}{{{\text{Xyl}}_{\text{in}} }} \times \frac{150}{96}$$

### Analysis of sugars

Pairs of aldoses and ketoses (i.e., glucose and fructose, xylose and xylulose) and furans (HMF and furfural) were analyzed separately by HPLC (Agilent 1100, Agilent Technologies, Palo Alto, CA) with an Aminex HPX87H Ion Exclusion column (300 × 7.8 mm, 8 µm particle size, Catalogue No. 125-0140) and Cation H+ guard column (BioRad Laboratories, Hercules, CA). The column parameters and operation conditions were the same as previously reported. 20 µL of sample was injected for analysis [7].

In mixed sugar experiments, the Aminex HPX87H column was unable to resolve all four aldoses and ketoses involved, so for analysis of these solutions, an anion chromatography system (Dionex ICS-5000, Dionex Corp., Sunnyvale, CA) was used. The column was a CarboPac PA-20 (3 × 150 mm, Dionex Corp.) operated at 35 °C. Optimal separation was achieved by isocratic elution at a flow rate of 0.5 mL min^−1^ with 20 mM potassium hydroxide (KOH) in 20 min. The KOH concentration was then raised to 100 mM and held at that concentration for an additional 5 min to regenerate the column. The column was then re-equilibrated at the starting eluent concentration for 10 min at the end of each run. The samples were diluted 1000-fold and injection volume was 10 µL. The sugar concentrations were measured with an ED50 electrochemical detector operated with the carbohydrate waveform and the data were analyzed using Dionex Chromeleon software (V 6.8).

## Results and discussions

### Enzymatic isomerization of glucose and xylose with and without borate

**Fig. 1:**
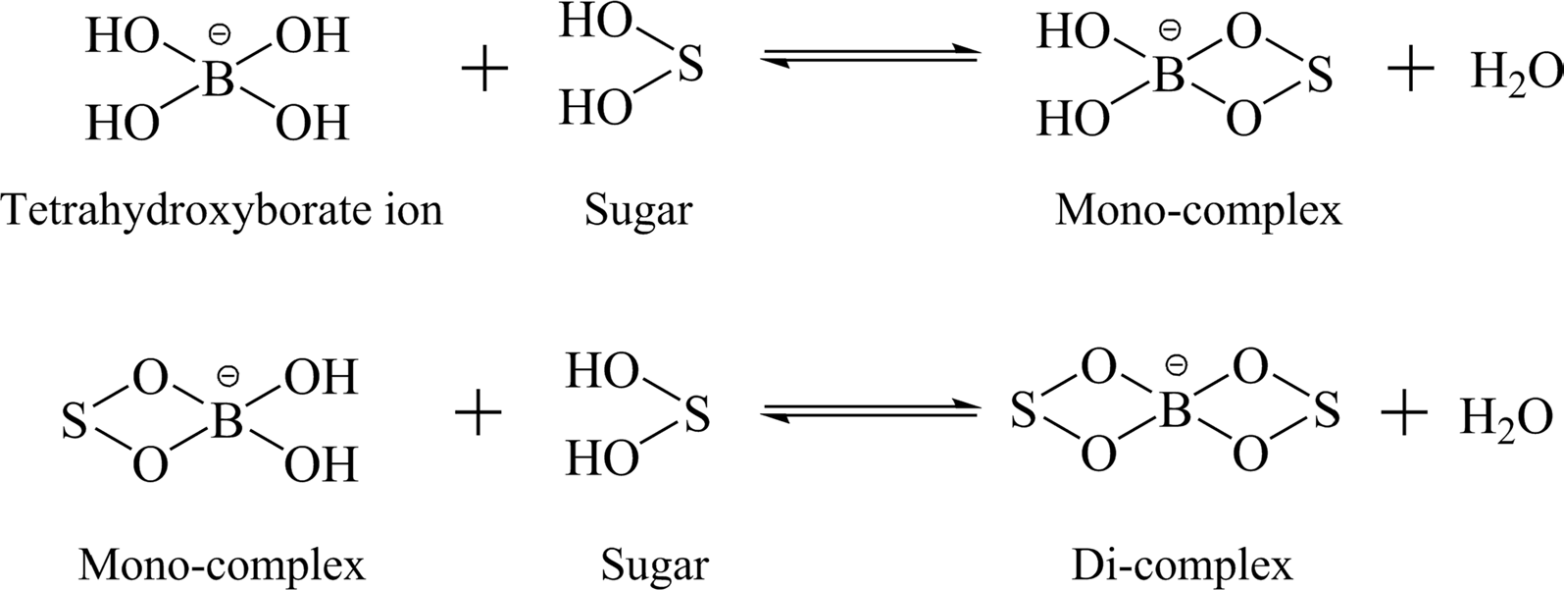
Mechanism for borate binding with two sugar molecules

The simultaneous isomerization of glucose and xylose to fructose and xylulose can be catalyzed by a bifunctional enzyme, i.e., a glucose isomerase. However, this reaction is reversible, which means there is a thermodynamic equilibrium in the isomerization between the aldose and ketose sugars. In this study, sodium tetraborate (borate) was used to shift the equilibrium to the ketoses. In aqueous solution, borate ions react with glucose and xylose to form ionized esters [22]. As shown in Fig. 1, each tetrahydroxyborate ion can bind with up to two molecules of sugar in a two-step process. Since borate has a higher affinity for fructose and xylulose than glucose and xylose, and the sugar–borate complexes do not participate in the isomerization reaction, the equilibria are shifted towards the ketoses in the isomerization reaction [13, 22–26].

**Fig. 2:**
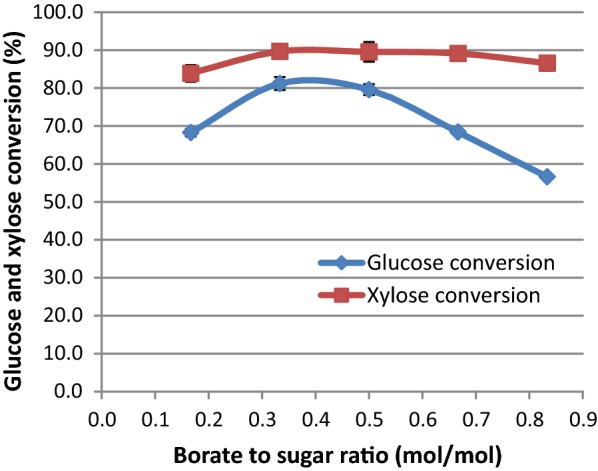
Effects of the molar ratio of borate to glucose and xylose on sugar isomerizations at 60 °C

Our results show that isomerization of the aldose sugars, i.e., glucose and xylose to fructose and xylulose, respectively, was higher when the borate to sugar molar ratio was in the range of 0.33 to about 0.5 (Fig. 2). Lower sugar isomerization levels were observed at lower and higher borate to glucose ratios. Based on our results and other reports, [13] we decided to use a borate to sugar molar ratio of 0.5 going forward unless otherwise noted.

**Fig. 3:**
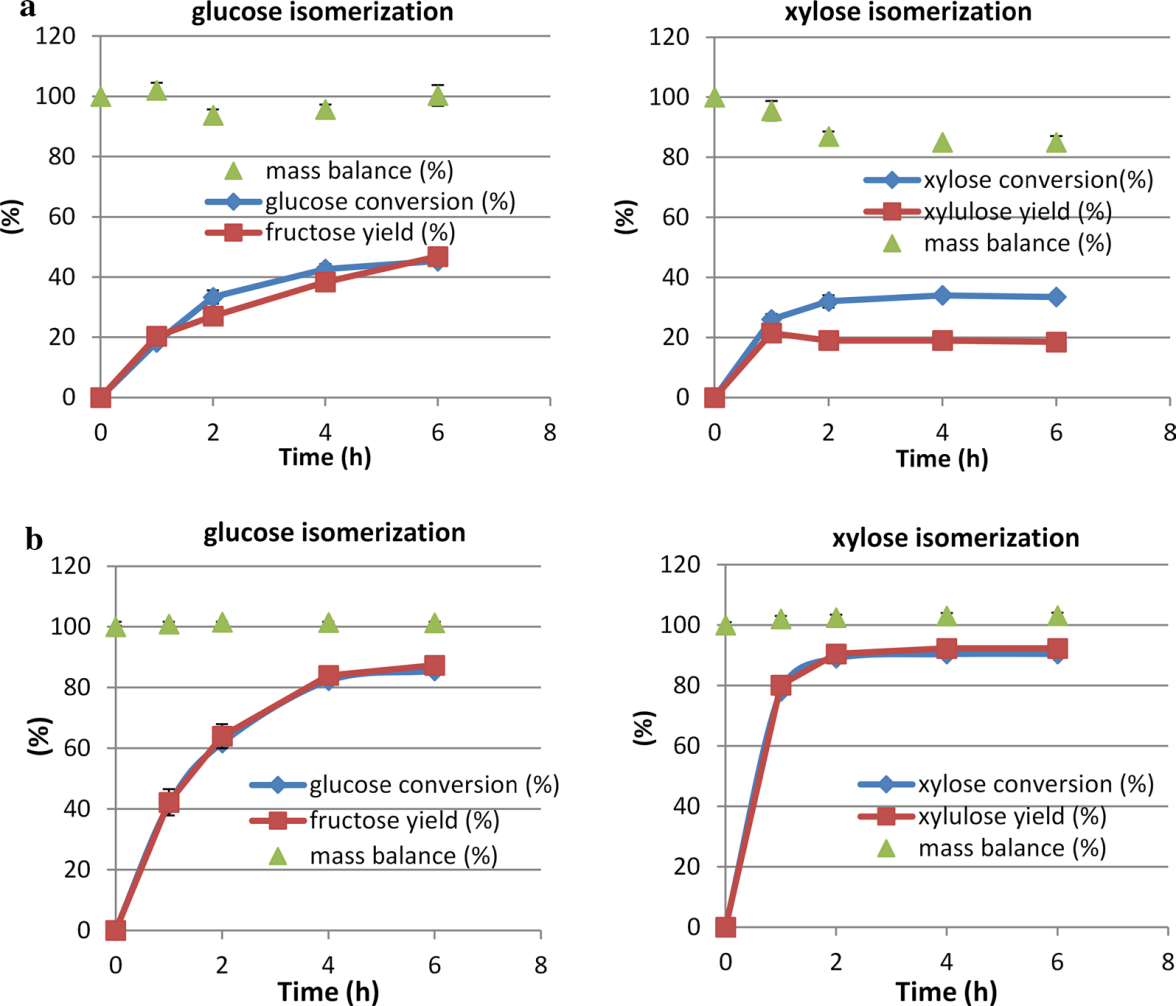
Isomerization of glucose (30 g/L) and xylose (20 g/L) without (**a**) and with (**b**) borate. Borate to sugar ratio 1:2, 60 °C

Figure 3 shows the separate isomerizations of glucose (30 g/L) and xylose (20 g/L) with and without borate at 60 °C. As is well known, glucose isomerase is used commercially for isomerizing glucose to fructose, to produce high fructose corn syrup, without the addition of borate, resulting in an approximately 50:50 mixture of glucose and fructose. In our isomerizations without borate, aldose conversions were incomplete and only low ketose yields were achieved (Fig. 3a). In the presence of borate, the rates of the isomerization reactions were much higher and more complete. With borate (Fig. 3b), xylose conversion reached 80% in 1 h and equilibrium after 2 h with a conversion of 91%. Compared to xylose, the rate of glucose isomerization even with borate was slower, reaching equilibrium after 4 h with a conversion of 84%. Our results proved that borate significantly enhanced the isomerization reactions and adding borate was essential to achieve high conversions and yields. Thereafter borate was used in all subsequent isomerization reactions in this study.

### Simultaneous isomerization of mixed sugars

**Fig. 4:**
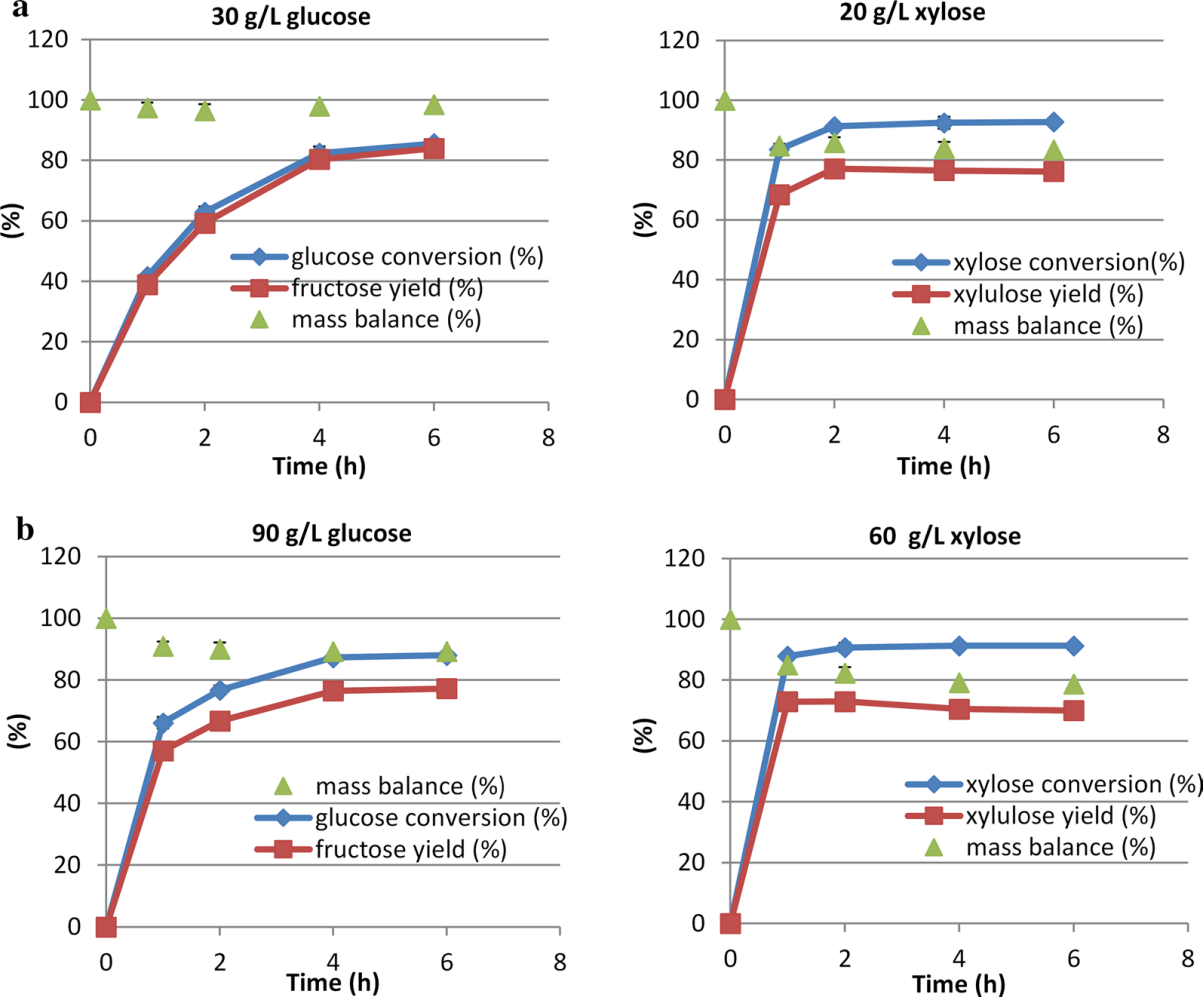
Isomerization of higher concentrations of mixed sugars (**b**) (90 g/L glucose and 60 g/L xylose) compared to low concentration sugars (**a**) (30 g/L glucose and 20 g/L xylose)

Simultaneous isomerization of mixed sugars (30 g/L glucose and 20 g/L xylose) in the presence of borate was investigated and the results are shown in Fig. 4a. It appears that mixing sugars together had little effect on sugar conversions. Glucose conversion was 80% after 4 h and xylose conversion reached 91% after 2 h, close to the conversions obtained in the isomerization of individual sugars (Fig. 3b). However, xylulose yield in mixed sugars was lower compared to that when isomerizing xylose alone, although the xylose conversion percentage was almost the same. This was possibly due to the persistence of the xylulose-borate complex which is stable under neutral and alkaline conditions. In the experiments with individual sugars the product solutions could be analyzed using a carbohydrate HPLC column that operates at acidic pH, however, this column was incapable of resolving all four carbohydrates (two aldoses and two ketoses) so that an anionic HPLC system (Dionex system) was used for analysis of the mixed sugars, which operates at alkaline pH. It is possible that a small amount of the xylulose-borate complex remained undetected when using the anionic HPLC system.

The overall goal of this study was to utilize biomass-derived sugars for upgrading to hydrocarbons. DMR hydrolysate from corn stover contains about 90 g/L glucose and 60 g/L xylose; therefore, we tested the sugar isomerization at higher concentrations. When isomerizing separate sugars at higher concentrations we observed slightly lower ketose yields. Fructose yield from 90 g/L of glucose was 77% compared to 83% from 30 g/L of glucose, and xylulose yield from 60 g/L of xylose was 70% compared to 76% from 20 g/L of xylose (Fig. 4b). However, glucose and xylose conversions were about the same at the higher concentrations as at the low sugar concentrations.

### Enzymatic isomerization without buffering

**Fig. 5:**
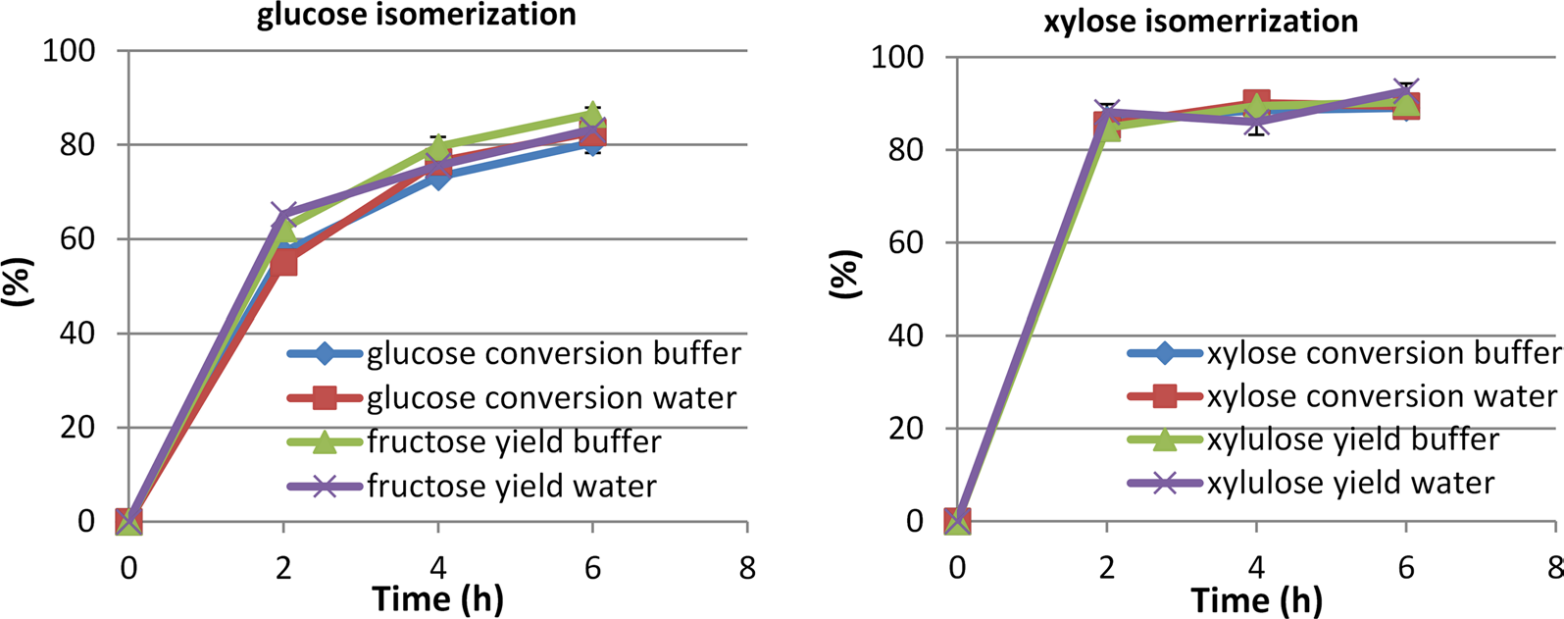
Isomerization of mixed sugars with and without buffer. Borate to glucose ratio 1:2, 60 °C

Buffering to keep a stable pH is important for stable enzyme activity. However, adding buffer leads to the use of much more acid in the following dehydration process for converting the sugars into furfurals. Thus, another issue that was investigated was the performance of the enzymatic isomerization with no added buffer. Results from this test indicated that, if no significant change in pH value occurred during incubation, the absence of the buffer had little effect on enzymatic isomerization in terms of aldose conversion and ketose yields, as shown in Fig. 5. Thus, the buffer was not used in subsequent aldose isomerization reactions.

### Simultaneous isomerization of sugars in DMR hydrolysate or dilute acid pretreated hydrolysate

**Fig. 6:**
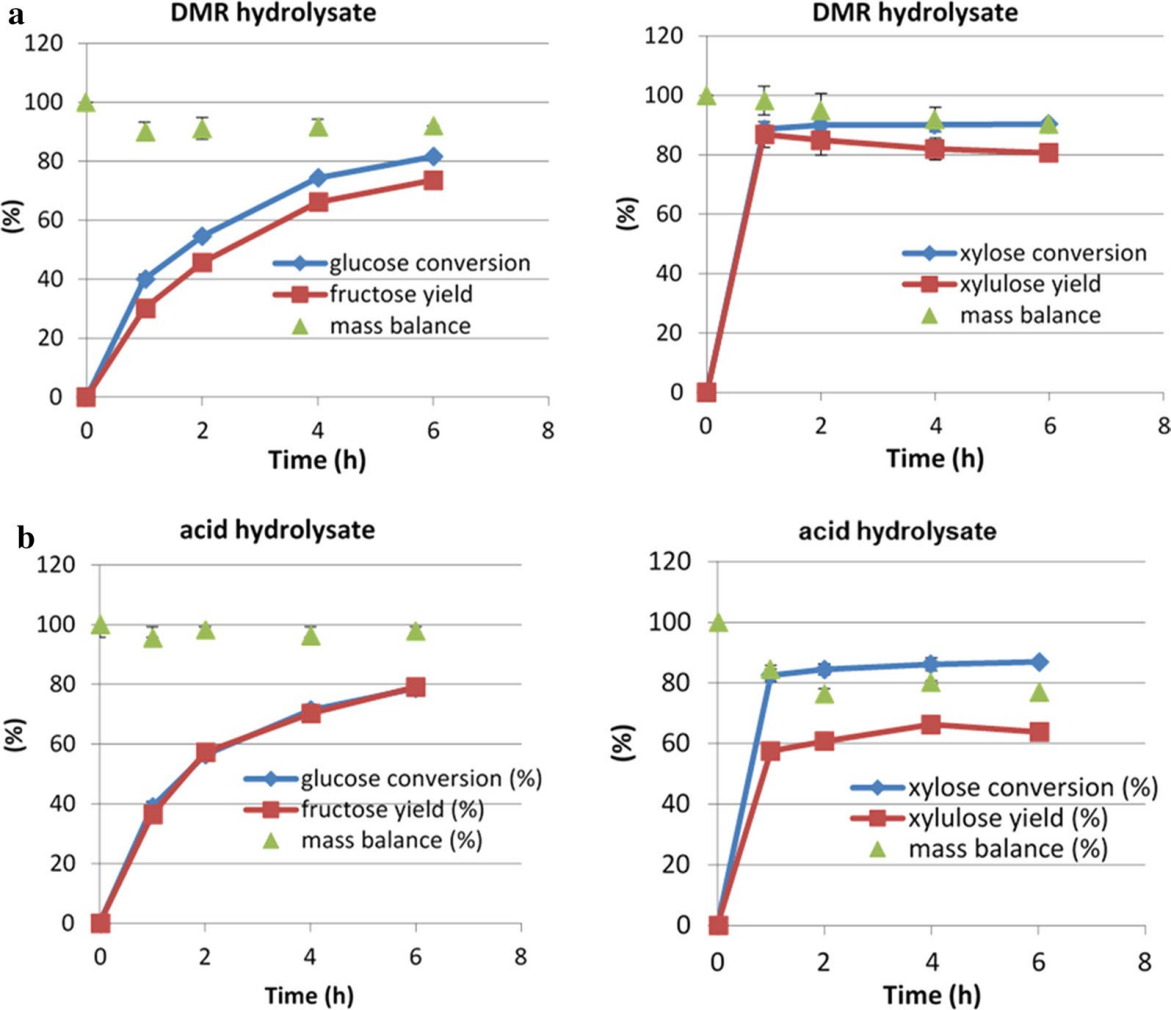
Enzymatic isomerization of DMR hydrolysate (**a**) or dilute acid hydrolysate (**b**). Borate to glucose ratio 1:2, 60 °C

The overall goal of this study was to utilize biomass-derived sugars for upgrading to hydrocarbons. In this context, after preliminary experiments with pure sugars, we started using hydrolysates made from the DMR process and dilute acid pretreated corn stover as feedstocks for enzymatic isomerization. The DMR hydrolysate used in this study was original, undiluted and unbuffered slurry with the pH adjusted to 7.5 by addition of sodium hydroxide. Isomerization experiments using DMR hydrolysate demonstrated that glucose conversion and fructose yield were slightly lower than with pure glucose while xylose conversion in the hydrolysate was close to that with pure xylose although xylulose yield was lower, as shown in Fig. 6a. These results show that impurities in DMR hydrolysate may affect the formation of sugar-borate complexes, as reflected by different ketose yields when compared to those from pure sugars, but the high conversion percentages of both sugars indicate DMR hydrolysate can be used as feedstock for sugar isomerization.

For dilute acid hydrolysate, as shown in Fig. 6b, no significant difference in performance was found between these DMR and acid hydrolysates in terms of glucose and xylose conversion, however, some differences in fructose and xylulose yields were observed, which could be due to the different amounts of sugar-borate complex in the two hydrolysates. These results demonstrated that both types of hydrolysate are good substrates for enzymatic isomerization considering the high sugar conversions.

### Production of furfural and HMF from mixed pure sugars

**Fig. 7:**
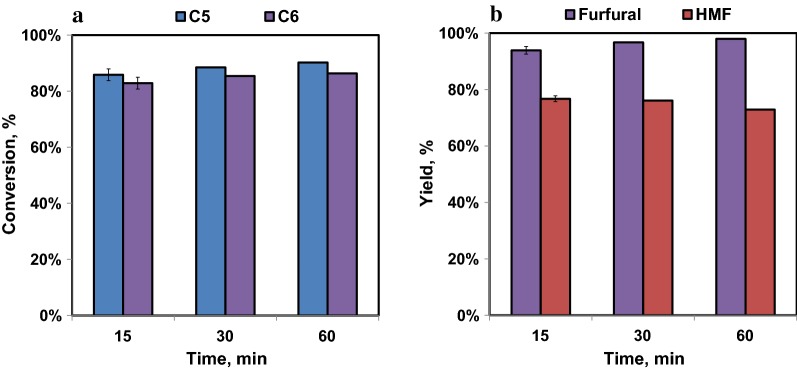
C5 and C6 conversion (**a**) and furfural and HMF production (**b**) as a function of reaction time at pH 0.5 obtained by dehydration of 90 g/L C6 (glucose and fructose) and 60 g/L C5 (xylose and xylulose) sugars conducted at 120 °C using HCl as the catalyst. Dioxane to aqueous ratio was 2:1. The error bars shown are standard deviations from triplicate analyses

Dehydration of ketose sugars was initially investigated using mixed pure sugars. Mixed glucose and xylose were isomerized first to ketoses followed by dehydration of the isomerized sugar solution. Figure 7 shows C5 and C6 sugar conversions, and furfural and HMF production from dehydration reactions conducted at 120 °C. The dioxane to aqueous ratio was 2:1, and HCl was used to obtain the optimal pH of 0.5.

High C5 sugar conversions (85% to 90%) were achieved in 15 to 60 min as shown in Fig. 7a. C6 sugar conversions (83% to 86%) were only slightly lower. High furfural and HMF yields (94 to 98% and 75 to 77%, respectively) were also observed. However, the maximum furfural yield (98%) was obtained at 60 min while the maximum HMF yield (77%), was achieved after only 15 min (Fig. 7b). This is likely due to the greater sensitivity of HMF to degradation reactions to humins and carboxylic acids. The reason we achieved higher furfural yields than C5 sugar conversions (theoretically furfural yields should be no more than 100%) can be attributed to the production of additional furfural, although in small quantities, from fructose dehydration.

### Furfural and HMF production from DMR hydrolysate

**Fig. 8:**
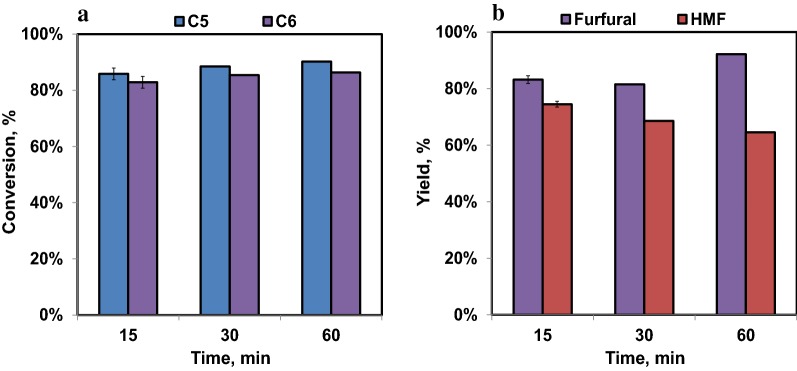
C5 and C6 conversion (**a**), and furfural and HMF production (**b**) as a function of reaction time obtained from corn stover DMR hydrolysate comprising 90 g/L C6 (glucose and fructose) and 60 g/L C5 (xylose and xylulose) sugars conducted at 120 °C and pH 0.5 obtained using HCl. Dioxane to aqueous ratio was 2:1. The error bars shown are standard deviations from triplicate analyses

Dehydration reactions were then performed on a DMR hydrolysate after the sugars had been isomerized to give a feed mostly containing ketoses. The reactions were conducted at the optimum temperature (120 °C) and pH (0.5) determined from the experiments using isomerized pure sugars, and reaction times of 15 to 60 min. The maximum furfural yield (92%) was obtained after 60 min, with a corresponding HMF yield of 65% (Fig. 8b). The maximum HMF yield (75%), however, occurred at 15 min with a corresponding furfural yield of 83%. This difference in the optimum reaction times for furfural and HMF production was also seen in the reaction of pure sugars and is again likely due to the greater sensitivity of HMF to degradation reactions. Overall, the furfural and HMF yields obtained from dehydration of DMR hydrolysate were slightly lower than the yields obtained with mixed pure sugars; the reason for this is unclear as it could be due to either a decrease in the efficiency of the isomerization reaction or in the dehydration reaction or both. The relatively low sugar conversions (Fig. 8a) are indicative of the presence of unconverted aldoses in the feeds, which do not react at the low temperature used for converting to ketoses. Comparing Figs. 7 and 8, it appears that the conversions were similar, so inhibition of the isomerization reaction is unlikely to completely explain the lower furfurals yields from the DMR hydrolysate. Consequently, it is at least possible that components of the DMR hydrolysate interfered with the dehydration reactions leading to the slight decreases in furfurals yields that were observed.

Similar experiments were performed on the C5 and C6 sugars in a dilute sulfuric acid hydrolysate and similar results were obtained (data not shown). Aldose conversions and furfural yields were about the same with the dilute acid hydrolysate as with the DMR hydrolysate.

## Conclusion

By isomerizing aldoses using a commercial immobilized enzyme and then dehydrating the resulting ketoses under acidic conditions, we have shown that furfural and HMF can be produced in high yields from the sugars in biomass hydrolysate. The yields of furfural and HMF from the sugars in biomass hydrolysates are only slightly lower than the yields coming from pure sugars, indicating that other components in the hydrolysate may have a slight effect on the conversion process. Isomerization of the aldoses permits dehydration reactions to be performed at much less severe conditions than those necessary for the conversion of the original aldoses, and at much higher yield. In the isomerization step, borate is necessary to push the equilibrium in the direction of the ketoses. Dioxane is needed as a co-solvent to get high yields of the furfurals. Future work will be aimed at trying to minimize the amounts of these chemicals used in the process and to calculate the cost of their use. Overall the significance of this work is that both the C5 and C6 sugars in a biomass hydrolysate, at a total initial sugar concentration of about 150 g/L, have been simultaneously converted to furfural and HMF in good yields after isomerization of the aldoses to ketoses.

## Data Availability

The datasets and materials in this study are available from the corresponding author on request.

## Abbreviations

HMF: 5-hydroxymethyl furfural
DMR: deacetylation and mechanical refining
HPLC: high performance liquid chromatography
